# The sterol carrier protein 2/3-oxoacyl-CoA thiolase (SCPx) is involved in cholesterol uptake in the midgut of *Spodoptera litura*: gene cloning, expression, localization and functional analyses

**DOI:** 10.1186/1471-2199-10-102

**Published:** 2009-11-13

**Authors:** Xing-Rong Guo, Si-Chun Zheng, Lin Liu, Qi-Li Feng

**Affiliations:** 1 Guangdong Provincial Key Lab of Biotechnology for Plant Development, School of Life Sciences, South China Normal University, Guangzhou, 510631, PR China; 2 The Faculty of Pharmacy and Laboratory Medicine, Yunyang Medical College, Hubei, 442000, PR China

## Abstract

**Background:**

Sterol carrier protein-2/3-oxoacyl-CoA thiolase (SCPx) gene has been suggested to be involved in absorption and transport of cholesterol. Cholesterol is a membrane component and is a precursor of ecdysteroids, but cannot be synthesized *de novo *in insects. However, a direct association between SCPx gene expression, cholesterol absorption and development in lepidopteran insects remains to be experimentally demonstrated.

**Results:**

An SCPx cDNA (*Sl*SCPx) cloned from the common cutworm, *Spodoptera litura*, was characterized. The *Sl*SCPx cDNA encoded a 535-amino acid protein consisting of a 3-oxoacyl-CoA thiolase (SCPx-t) domain and a SCP-2 (SCPx-2) domain. *Sl*SCPx mRNA was expressed predominately in the midgut, while *Sl*SCPx-2 mRNA was detected in the midgut, fat body and epidermis and no *Sl*SCPx-t mRNA was detected. A 58-kDa full-length SCPx protein and a 44-kDa SCPx-t protein were detected in the midgut of sixth instar larvae when the anti-*Sl*SCPx-t antibody was used in western blotting analysis; a 16-kDa SCP-2 protein was detected when anti-*Sl*SCPx-2 antibody was used. *Sl*SCPx protein was post-translationally cleaved into two smaller proteins, SCPx-t and SCPx-2. The gene appeared to be expressed into two forms of mRNA transcripts, which were translated into the two proteins, respectively. *Sl*SCPx-t and *Sl*SCPx-2 proteins have distinct and different locations in the midgut of sixth instar larvae. *Sl*SCPx and *Sl*SCPx-t proteins were detected predominately in the cytoplasm, whereas *Sl*SCPx-2 protein was detected in the cytoplasm and nuclei in the Spli-221 cells. Over-expression of *Sl*SCPx and *Sl*SCPx-2 proteins enhanced cholesterol uptake into the Spli-221 cells. Knocking-down *Sl*SCPx transcripts by dsRNA interference resulted in a decrease in cholesterol level in the hemolymph and delayed the larval to pupal transition.

**Conclusion:**

Spatial and temporal expression pattern of this *Sl*SCPx gene during the larval developmental stages of *S. litura *showed its specific association with the midgut at the feeding stage. Over-expression of this gene increased cholesterol uptake and interference of its transcript decreased cholesterol uptake and delayed the larval to pupal metamorphosis. All of these results taken together suggest that this midgut-specific *Sl*SCPx gene is important for cholesterol uptake and normal development in *S. litura*.

## Background

Sterol carrier protein 2/3-oxoacyl-CoA thiolase (SCPx) belongs to a well-characterized SCP-2 gene family [1], whose members encode an intracellular non-specific lipid carrier protein. SCP-2 is present in both vertebrates and invertebrates and is involved in intracellular sterol/lipid transfer processes, which affect biosynthesis and metabolism of fatty acids and sterols [2]. In insects, cholesterol is required for cellular membranes and ecdysteroid biosynthesis. Insects utilize phytols, such as β-sitosterol, campesterol and stigmasterol, and synthesize ecdysteroids (molting hormone) in the prothoracic glands [3]. However, insects cannot synthesize cholesterol via *de novo *biosynthesis because they lack at least two key enzymes, squalene monooxygenase and lanosterol synthase, in their system [4,5]. Therefore insects must obtain cholesterol or other sterols from their diet to fulfill their sterol requirements for normal growth, development and reproduction [1,6-8]. In humans [9], mice [10], rats [11] and chickens [12], a single SCPx gene encodes a fusion protein containing 3-oxoacyl-CoA thiolase (SCPx-t) and SCPx-2 domains, which are post-translationally cleaved into two separate proteins. The SCPx-t protein functions as a 3-oxoacyl-CoA thiolase in peroxisomal oxidation of branched-chain fatty acids [13]. The SCP-2 protein is released from the peroxisomes into the cytoplasm and then translocated into the nucleus, where it acts as a transcription factor [14]. This gene is also transcribed into a transcript that encodes only the SCP-2 protein depending on alternative transcription initiation [9-12,15,16]. In invertebrates, members of the SCP-2 gene family have been reported in many species. In *Caenorhabditis elegans*, the genes encoding 3-oxoacyl-CoA thiolase (SCPx-t) and SCP-2 protein are not fused together and the two proteins are encoded by separate genes, P44, which is a thiolase-type protein homologous to the *N*-terminal protein SCPx-t of the vertebrate SCPx, and UNC-24, which is homologous to the *C*-terminal SCPx-2 protein of the vertebrate SCPx [17,18]. In *Aedes aegypti *and *Drosophila melanogaster *the SCPx genes encode a SCPx transcript of mRNA that encodes both SCPx-t and SCPx-2 domains [19,20], while there are separate genes producing other low-molecular-mass SCP-2 proteins in *A. aegypti *[2]. In the lepidopteran insects *Bombyx mori *and *Spodoptera littoralis*, the SCPx gene also encodes two fused SCPx-t and SCP-2 domains [21,22].

SCPx deletion mutant mice accumulated a derivative of the intermediate 24-keto-trihydroxy cholestanoic acid-CoA (24-keto-THCA-CoA), suggesting that the products of the SCPx gene are responsible for the cleavage of 24-keto-THCA-CoA into choloyl-CoA [13]. Over-expression of SCPx in mouse L-cells significantly altered cholesterol absorption and metabolism [23]. Knocking down *Ae*SCP-2 transcript decreased the accumulated level of cholesterol in the pupae and resulted in increased mortality of the mosquito *A. aegypti *adults, indicating that the *Ae*SCP-2 gene is critical for adult development [24]. In transfected mouse L-cells SCPx/SCP-2 co-localized with catalase in peroxisomes, but significant amounts of SCPx/SCP-2 appeared to be extra-peroxisomal [1,23]. In both *in vitro *cultured cells and the larval midgut of *A. aegypti*, *Ae*SCPx was present mostly in the peroxisomes, while *Ae*SCP-2, which is not transcribed from *Ae*SCPx gene in *A. aegypti *cells [20], was present in the cytosol, mitochondria and nuclei [25]. The difference in the subcellular distribution of SCPx and SCPx-2 suggests that these two proteins may play different and specific roles in cellular processes.

In the present study, cloning, characterization, cellular localization and functional analysis of a SCPx cDNA in *Spodoptera litura *were described. *Sl*SCPx is predominately expressed in the midgut during the larval feeding stage. Knocking down *Sl*SCPx transcripts in larvae and over-expressing *Sl*SCPx and *Sl*SCPx-2 in the Spli-221 cell line altered the accumulation of cholesterol and adversely affected the larval to pupal transition. All of these results taken together suggest that the *Sl*SCPx gene plays an important role in cholesterol uptake as well as growth and development of *S. litura*.

## Results

### Cloning and characterization of *Sl*SCPx cDNA sequence

**Figure 1:**
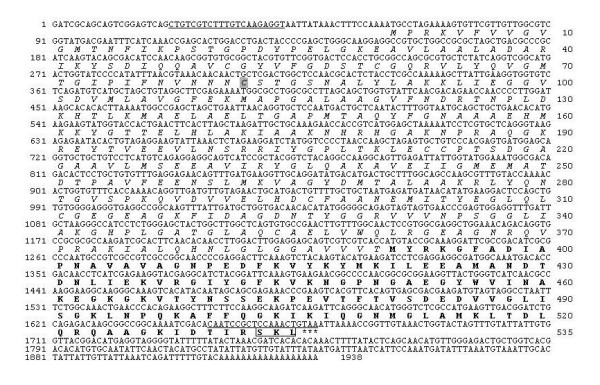
**Nucleotide and deduced amino acid sequences of *S. litura Sl*SCPx cDNA**. The numbers on the left are for nucleotide sequence and the numbers on the right are for the amino acid sequences. The thiolase domain (*Sl*SCPx-t) is shown in Italy letters and the *Sl*SCPx-C (SCP2) domain is indicated by bolded letters. The peroxisome targeting sequence (SKL) is indicated in a square. The forward and reverse primers used for RT-PCR amplification of the full length cDNA from mRNA are underlined. A cysteine residue (Cys82) is shown in grey background, which has been suggested to be part of the active site in a *Z. ramigera *3-oxoacyl-CoA thiolase. The terminal codon is indicated with asterisks below the codon. The GenBank accession number of this cDNA is FJ986464.

**Figure 2:**
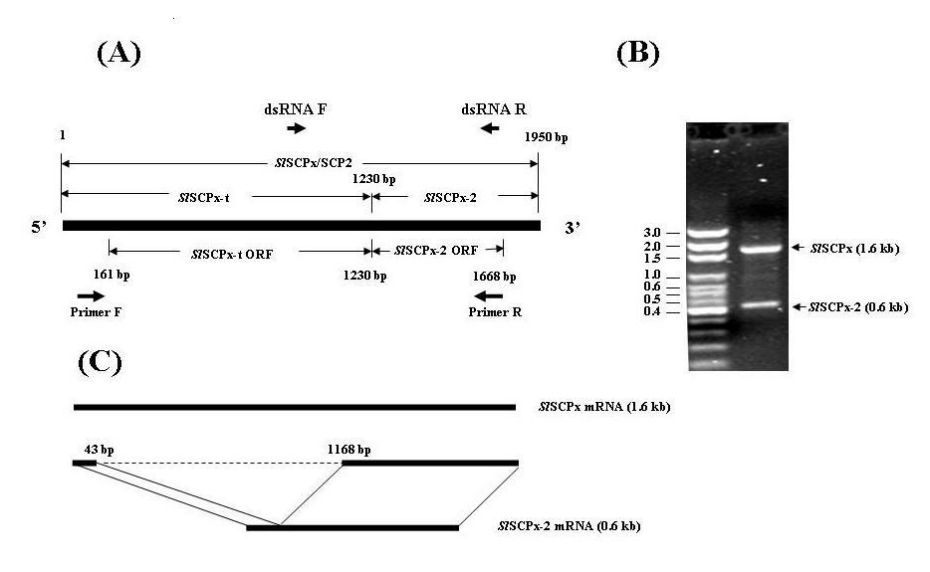
**RT-PCR amplification strategy of *S. litura Sl*SCPx cDNA from mRNA**. (A) RT-PCR amplification strategy, PCR-amplified products (B) and diagram of alternative splicing of *Sl*SCPx transcripts (C). The positions of the forward and reverse primers for RT-PCR are indicated in arrows below the sequence diagram. The arrows above the sequence diagram show the position of primers for the *Sl*SCPx dsRNA preparation (A). The two PCR products were amplified and their sizes were 1.6 kb for the full-length *Sl*SCPx and 0.6 kb for the *Sl*SCPx-C region, respectively (B). The dotted line shows the deleted *Sl*SCPx-t fragmentfrom the *N*-terminal (43 to 1,168 nucleotides) of *Sl*SCPx sequence, as compared to *Sl*SCPx-2.

A SCPx cDNA sequence was identified by randomly picking and sequencing cDNA clones in an expression library constructed with mRNA isolated from the midgut of 3-day-old 6^th ^instar larvae of *S. litura*. This cDNA (*Sl*SCPx) contained 1,938 bp in length encoding an open reading frame (ORF) of 535-amino acids with an estimated molecular mass of 58 kDa, which included two domains, one containing 390-amino acid residues with an estimated molecular mass of 41 kDa at the *N*-terminal end and the other containing 145-amino acid residues with an estimated molecular mass of 16 kDa at the *C*-terminal end (Fig. 1). Blast search in the GenBank database indicated that the first domain was highly homologous to 3-oxoacyl-CoA thiolase, while the second domain was highly homologous to SCP-2 protein in other species. For the sake of convenience in this paper, the full-length protein is referred to as *Sl*SCPx; the 3-oxoacyl-CoA thiolase domain is referred to as *Sl*SCPx-t; and the SCP-2 domain is referred to as *Sl*SCPx-2 (Fig. 1). To confirm and obtain the *Sl*SCPx full-length cDNA directly from the *S. litura *midgut, reverse-transcription PCR (RT-PCR) was performed using a pair of primers designed on the basis of the cloned *Sl*SCPx cDNA sequence (Fig. 1 and 2A) and mRNA extracted from the midgut of 3-day-old 6^th ^instar larvae. Interestingly, two PCR products of approximately 1.6 kb and 0.6 kb were obtained (Fig. 2B). The results of sequencing and Blast search indicated that they encoded a full-length SCPx protein, which included both SCPx-t and SCPx-2 domains, and a SCP-2 protein, respectively. Sequence comparison of the *Sl*SCPx cDNA with the sequences of the two PCR products revealed that the PCR product SCPx (1.6 kb) was identical to the *Sl*SCPx full-length cDNA cloned from the screening of the library, while the SCPx-2 cDNA (0.6-kb) was identical to the *C*-terminal *Sl*SCPx-2 domain of the *Sl*SCPx cDNA, but lacked the *N*-terminal SCPx-t sequence between the 43 and 1,168 nucleotides of the sequence. Similar results were observed for transcripts of SCPx/2 gene in *B. mori*, where two transcripts approximately 1.7- and 0.6-kb cDNA products were amplified by RT-PCR [22].

The deduced *Sl*SCPx protein shared the same domain architecture as that of the vertebrate SCPx: a thiolase (SCPx-t) at the *N*-terminal region and a SCPx-2 domain at the *C*-terminal region (Fig. 1). The *C*-terminal regions of the *Sl*SCPx protein and the *Sl*SCPx-2 protein contained a peroxisomal-targeting signal Ser-Lys-Leu. It is likely that *Sl*SCPx and *Sl*SCPx-2 proteins can be transported to peroxisomes where they perform their functions. A cysteine residue (Cys82) (Fig. 1A), corresponding to the catalytically active cysteine in *Zoogloea ramigera *3-ketoacyl-CoA thiolase [26], was conserved in the *Sl*SCPx protein.

**Figure 3:**
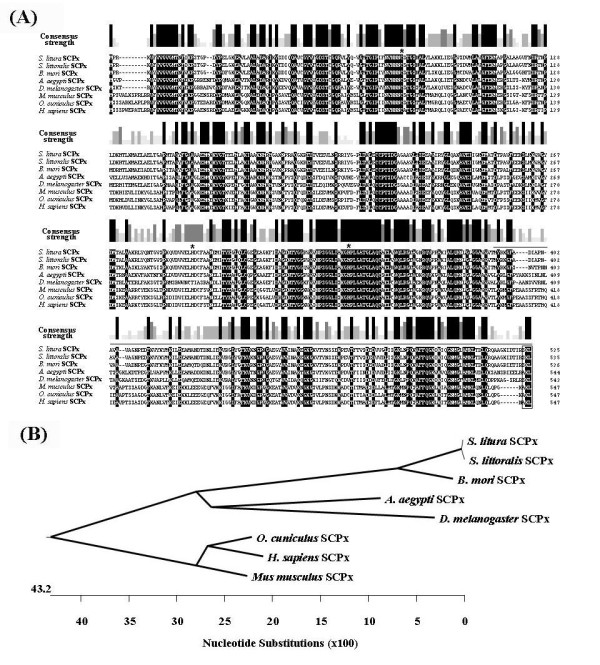
**Alignment and phyologenetic tree analysis of the deduced amino acid sequences of *Sl*SCPx and its homologues**. (A) Sequence alignment and (B) phyologenetic analysis of SCPx homologues from other species. *S. littoralis *SCPx (AAT72922), *A. aegypti *SCPx (AAF53713), *B. mori *SCPx (NP_001037378), *D. melanogaster *SCPx (NP_524715), *O. cuniculus *SCPx (AF051897), *H. sapiens *SCP-x (P32020) and *M. musculus *SCPx (AAA40098). The amino acid residues that are identical are indicated by black background. The consensus strength is shown above the sequence alignment panels. The three amino acid residues required for reaction activity of thiolases are indicated by asterisks. The peroxisome targeting sequence (SKL or AKL) is boxed. The SCPx-2 region is indicated by a line above the sequence panel.

Blast search analysis indicated that *Sl*SCPx protein was a member of the SCP-2 gene family and highly similar to SCPx in other species, such as SCPx (99%) of *S. littoralis *Boisduval [21], which is a geographically isolated allopatric species of *S. litura *Fabricius (CABI and EPPO, 1990), *B. mori *SCPx (88%) [22], *A. aegypti *SCPx (60%) [20], *D. melanogaster *SCPx (63%) [19], *O. cuniculus *SCPx (55%) [27], *Mus musculus *SCPx (55%) [23] and *Homo sapiens *SCPx (54%) [9] (Fig. 3A). This indicates that this gene is well-conserved during evolution and it may play a conserved functional role in growth and development of insects. Although the lepidopteran SCPx have higher identities to the dipteran SCPx in regard to their amino acid sequence and they are clustered into an insect group, the lepidopteran SCPx appeared to be orthologs of the vertebrate SCPx (Fig. 3B).

### *In vitro *expression of *Sl*SCPx, *Sl*SCPx-t, *Sl*SCPx-2 recombinant proteins

**Figure 4:**
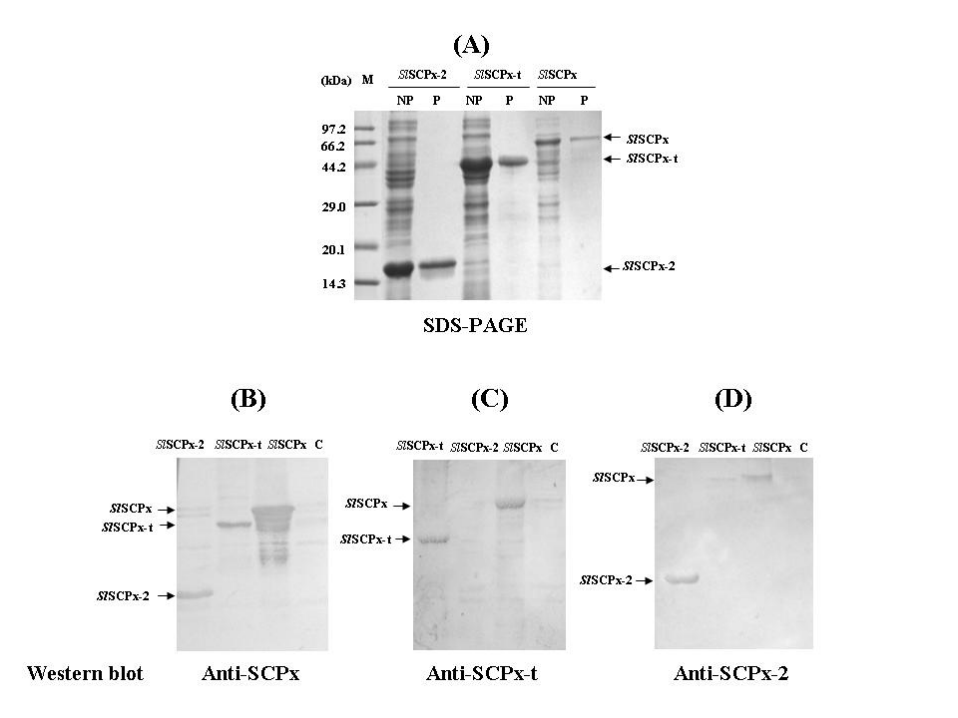
***In vitro *expression, purification and antibody production of *Sl*SCPx, *Sl*SCPx-t and *Sl*SCPx-2 recombinant proteins in a bacterial expression system**. (A) *In vitro *expression and purification of the recombinant proteins; (B-D) immune reaction of *Sl*SCPx, *Sl*SCPx-t and *Sl*SCPx-2 recombinant proteins. The recombinant proteins were purified using the His-tag affinity column. The protein samples (10 μg/lane) were analyzed on 15% SDS-PAGE gel and stained with Commassie Blue R-250. On the western blotting analyses (B-D) the proteins were blotted with the corresponding anti-*Sl*SCPx antiserium at 1:1,500 dilution. C: control; NP: non-purified; P: purified.

*In vitro *expression of the *Sl*SCPx (full-length protein), *Sl*SCPx-t (the SCPx-t domain at the *N*-terminus) and *Sl*SCPx-2 (the SCP-2 domain at the *C*-terminus) proteins respectively were achieved in a bacterial expression system and the recombinant His-tagged fusion proteins were purified using His-tag affinity columns (Fig. 4A). The resultant proteins had the identical molecular mass as predicted based on the deduced amino acid sequences. Antibodies against these three peptides, respectively, were generated and could specifically recognize and distinguish the recombinant proteins from each other in western blotting analyses (Fig. 4B-D).

### Temporal and spatial expression of *Sl*SCPx gene

**Figure 5:**
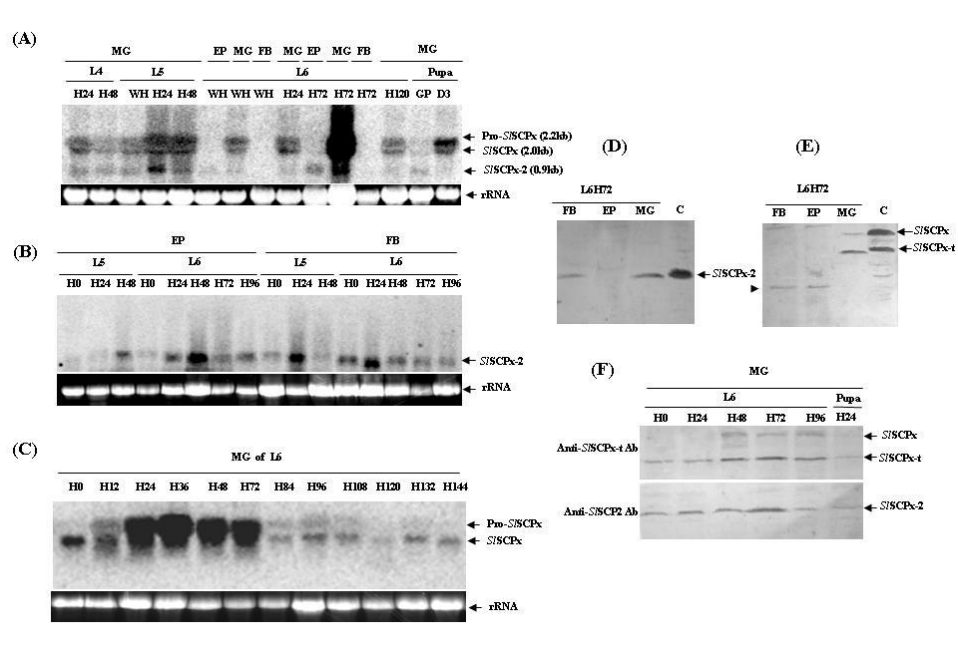
**Northern and western blotting analysis of temporal and spatial expression of the *Sl*SCPx gene and protein**. Ten microgram aliquots of total RNA from various tissues and stages was used in each of the lanes in the northern blots (A-C). The blots were hybridized with ^32^P-labelled DNA probes of the *Sl*SCPx-2 region (A and B) and the *Sl*SCPx-t region (C), respectively. In the western blots (D-F), the protein extracts were blotted with anti-*Sl*SCPx-2 antibody (D and the lower panel of F) and anti-*Sl*SCPx-t antibody (E and the upper panel of F), respectively. The arrowhead in (E) indicates an unidentified protein present in the fat body and epidermis. C: the recombinant protein extract from bacterial expression system. L: larvae; D: day; H: hours; WH: white head; MG: midgut; EP: epidermis; FB: fat body; P: pupae. Pro-*Sl*SCPx is the putative pre-mutual transcript of the full-length *Sl*SCPx mRNA.

When a DNA fragment at the *Sl*SCPx-2 domain region of the *Sl*SCPx gene was used as a probe, three mRNA transcripts (2.8, 2.0 and 0.9 kb) were detected in the midgut of different stages (Fig. 5A). They appeared to be the transcripts encoding *Sl*SCPx (2.8 kb and 2.0 kb) and *Sl*SCPx-2 (0.9 kb) mRNA, respectively. The 2.8-kb and 2.0-kb transcripts of *Sl*SCPx were probably the pre-mature and mature mRNA products of the gene, respectively [23] and they were highly and specifically expressed in the midgut (Fig. 5A). In contrast, the *Sl*SCPx-2 mRNA (0.9 kb) was also detected in the fat body and epidermis in addition to the midgut during larval and pupal development (Fig. 5A and 5B). In the midgut, the expression of the *Sl*SCPx transcripts was much higher during the larval feeding stages (for example, days 1 to 2 of 5^th ^instar and days 1 to 3 of 6^th ^instar larvae) than during the larval molting (L6 white head) stage and the larval to pupal transition (green pupae) (Fig. 5A). The expression declined to a lower level after day 2 post pupation. The *Sl*SCPx-2 (0.9 kb) mRNA was also detected in the epidermis and fat body when a labelled *Sl*SCPx-2 DNA fragment was used as a probe (Fig. 5B). When a labelled DNA fragment of the *Sl*SCPx-t region was used as a probe, only the *Sl*SCPx (2.8 kb and 2.0 kb) mRNA transcripts were detected (Fig. 5C). These two transcripts were highly expressed during the feeding stage (days 1 to 3) of 6^th ^instar larvae, but decreased when the larvae started wandering and entered the prepupal stage (Fig. 5C). These northern blotting analyses indicated that *Sl*SCPx was specifically and highly expressed in the midgut during the larval feeding stage, while *Sl*SCPx-2 mRNA was also found in the epidermis and fat body, in addition to the midgut.

Western blotting analysis was performed to examine the levels of these proteins in the different tissues and stages of 6^th ^instar larvae (Fig. 5D-F). The anti-*Sl*SCPx-2 antibody detected a single band of *Sl*SCP-2 protein in the midgut and fat body of 3-day-old 6^th ^instar larvae, but not in the epidermis (Fig. 5D). This was contrast to the northern blotting analysis where the *Sl*SCPx-2 transcript was also detected in the epidermis (Fig. 5A). This is probably because only a very low level of *Sl*SCPx-2 protein translation occurred in the epidermis at this stage. The anti-*Sl*SCPx-t antibody detected a major 44-kDa protein and a weak 58-kDa protein, which were closely equivalent to the predicted molecular mass of the *Sl*SCPx-t (thiolase domain) and full-length *Sl*SCPx proteins, respectively and as observed for bacterially expressed equivalents (Fig. 5E). No such proteins were detected in the fat body and epidermis, where another small unidentified protein immunologically reacted with the anti-*Sl*SCPx-t antibody. These results indicated either that *Sl*SCPx was post-translationally and proteolytically cleaved into two smaller proteins *Sl*SCPx-t and *Sl*SCPx-2, or that the transcription of the *Sl*SCPx gene was initiated at two different transcription initiation sites, generating a full-length *Sl*SCPx mRNA, which was translated into a full-length protein that was proteolytically cleaved into *Sl*SCPx-t and *Sl*SCPx-2 proteins post translationally, and a *Sl*SCPx-2 mRNA that was translated into a *Sl*SCPx-2 protein. It was also noticed that the relative levels of *Sl*SCPx-t and *Sl*SCPx-2 proteins were higher than that of *Sl*SCPx protein, suggesting that *Sl*SCPx protein was rapidly cleaved into *Sl*SCPx-t and *Sl*SCPx-2 proteins after synthesis. The levels of the three proteins *Sl*SCPx, *Sl*SCPx-t, *Sl*SCPx-2 were higher during the feeding stage (for example, days 1-3 of 6^th ^instar) than non-feeding stages (for example, day 0 of 6^th ^instar larval stage and pupal stage) (Fig. 5F). These results from protein analysis were consistent with the results of northern blotting analyses for mRNA profiles.

### Tissue and cellular localization of *Sl*SCPx, *Sl*SCPx-t and *Sl*SCPx-2 proteins in larvae and *in vitro *Spli-221 cells

**Figure 6:**
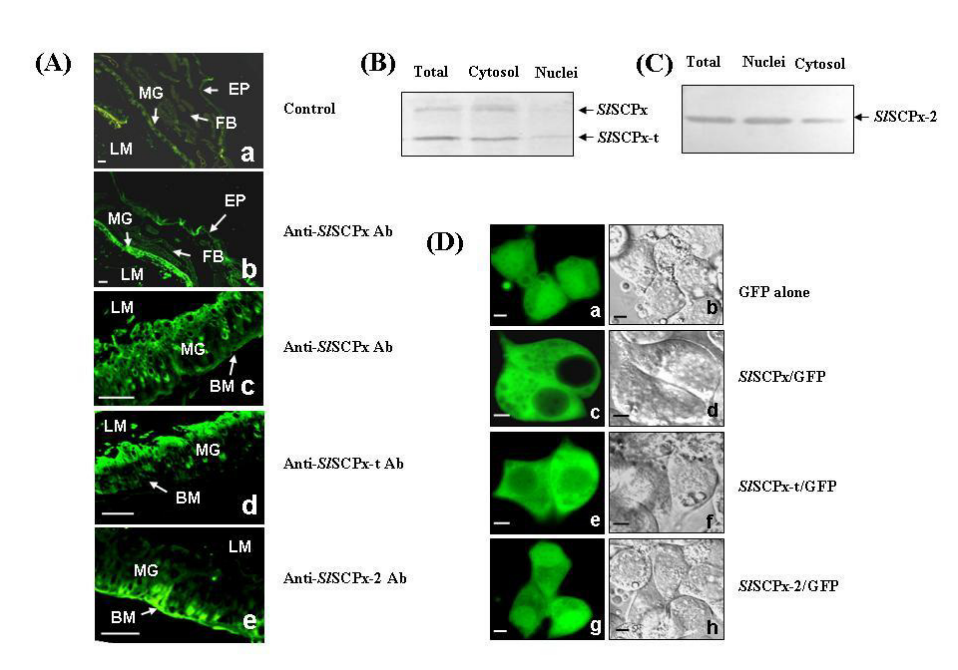
**Localization of *Sl*SCPx, *Sl*SCPx-t and *Sl*SCPx-2 proteins**. (A) Immunohistochemistry localization of *Sl*SCPx, *Sl*SCPx-t and *Sl*SCPx-2 proteins in the 2-day-old 6^th ^instar larvae of *S. litura*; (B and C) subcellular localization of the proteins in epithelial cells of the midgutand in the transfected Spli-221 cells (D). Anti-*Sl*SCPx, *Sl*SCPx-t and *Sl*SCPx-2 antibodies were used at a dilution of 1:200 in (A). The proteins for western blotting analysis were extracted from the cytoplasm and nuclei of the midgut epithelial cells of 3-day-old old 6^th ^instar larvae. Following electrophoresis of 20 μg protein/lane blots were probed by anti-*Sl*SCPx-t (B) and anti-*Sl*SCPx-2 (C) antibodies, respectively. The bands in (B) run at approximately 58 kDa and 44 kDa, respectively, and the band in (C) at approximately 16 kDa. (D) Spli-221 cells were transfected with pEGFP1 (Da and Db), pE*Sl*SCPx/GFP (Dc and Dd), pE*Sl*SCPx-t/GFP (De and Df) and pE*Sl*SCPx-C/GFP (Dg and Dh) plasmid DNAs, respectively. The photographs were taken through fluorescence (Da, Dc, De and Dg) and visible light filters (Db, Dd, Df and Dh) at 24 h post transfection. MG: midgut; EP: epidermis; FB: fat body; LM: lumen; BM: basal membrane. The scale bars represent 60 μm in (A) and 10 μm in (D).

Localization of *Sl*SCPx, *Sl*SCPx-t and *Sl*SCPx-2 proteins in larvae was examined using immunohistochemistry (Fig. 6). When anti-*Sl*SCPx antibody was used as the primary antibody, the protein was detected mainly in the midgut of 2-day-old 6^th ^instar larvae (Fig. 6Ab), although a trace amount of protein was also found in the epidermis and fat body. This result was consistent with the result of the western blotting analysis, in which the protein was mainly present in the midgut (Fig. 5D-F). In addition, the *Sl*SCPx, *Sl*SCPx-t and *Sl*SCPx-2 proteins were distributed in the different regions of the midgut epithelium (Fig. 6Ab-d). The *Sl*SCPx protein was distributed almost evenly in most of the epithelial cells (Fig. 6Ac), while the *Sl*SCPx-t protein was detected mostly in the epithelial cells that were close to the lumen side (Fig. 6Ad) and the *Sl*SCPx-2 protein was localized in the epithelial cells that were close to the basal membrane side of the midgut epithelium (Fig. 6Ae). Subcellular distribution of the proteins was also examined by using two approaches. Firstly, protein extracts were isolated from the cytosol and nuclear fractions, respectively, from the midgut epithelial cells of 3-day-old 6^th ^instar larvae and were analyzed by western blots using anti-*Sl*SCPx-t and anti-*Sl*SCPx-2 antibodies (Fig. 6B and 6C). The anti-*Sl*SCPx-t antibody detected two protein bands (approximately 58 kDa and 44 kDa), presumably being *Sl*SCPx and *Sl*SCPx-t, respectively, at a higher level in the cytoplasm than in the nuclei of the epithelial cells (Fig. 6B), indicating that these two proteins were mainly present in the cytoplasmic fraction. The anti-*Sl*SCPx-2 antibody detected the protein at a higher level in the nuclei than in the cytosplasm of the midgut epithelial cells (Fig. 6C), suggesting that the *Sl*SCPx-2 protein was primarily in the nuclei. Secondly, the embryogenic cell line, Spli-221, of *S. litura *was transfected with the eukaryotic expression vector pEGFP-1 expressing *Sl*SCPx/GFP, *Sl*SCPx-t/GFP and *Sl*SCPx-2/GFP fusion proteins, respectively (Fig. 6D). At 24 h post transfection, more than 60% of the cells expressed green fluorescence, indicating that most of the cells were transfected by the plasmid DNAs. The confocal fluorescence microscopy observation on the transfected cells revealed that the green fluorescence signal in the control cells, which were transfected with the plasmid pEGFP-1 expressing GFP alone, was seen in both the cytoplasm and nuclei (Fig. 6Da). In the *Sl*SCPx/GFP over-expressing cells the green fluorescence was detected only in the cytosol and almost no signal was found in the nuclei (Fig. 6Dc). In the *Sl*SCPx-t/GFP over-expressing cells, stronger fluorescence signal was found in the cytoplasm than in the nuclei (Fig. 6De). The fluorescence signal of *Sl*SCPx-2/GFP was also seen in both the cytoplasm and nuclei, but with a higher intensity in the nuclei than in the cytoplasm (Fig. 6Dg). All of these results from the immunohistochemistry of the midgut epithelial tissues (Fig. 6A), western blotting analyses of cytoplasmic and nuclear protein extracts (Fig. 6B and 6C), and transient expression of the GFP-marked proteins in the cultured Spli-221 cells (Fig. 6D), taken together suggest that the *Sl*SCPx and *Sl*SCPx-t proteins are localized predominately in the cytoplasm of the epithelial cells, particularly the cells that are close to the lumen side of the epithelium in the case of *Sl*SCPx-t protein, while the *Sl*SCPx-2 protein was localized in both the nuclei and the cytoplasm of the epithelial cells that were close to the basal membrane side of the midgut epithelium.

### Effect of *Sl*SCPx and *Sl*SCPx-2 expression on cholesterol uptake in the Spli-221 cell line

**Figure 7:**
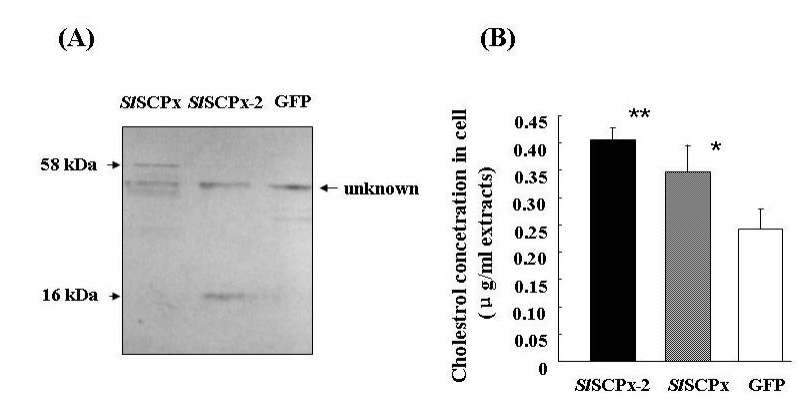
**Effect of *Sl*SCP-x and *Sl*SCPx-2 protein expression on the absorption of cholesterol in the Spli-221 cells transfected with pE*Sl*SCPx/GFP and pE*Sl*SCPx-2/GFP transfection vectors, respectively**. (A) Western blotting analysis of the protein extracts from the transfected Spli-221 cells. Proteins were analyzed on a 12% SDS-PAGE gel and each lane contained 20 μg protein. (B) Cholesterol concentrations in the transfected cells were analyzed using HPLC at 36 h post transfection. The control was the cells transfected with a pEGFP vector expressing only GFP. Three independent replicates were performed for each data point. The concentration of cholesterol in the cells or hemolymph was calculated as μg/ml of medium or hemolymph.

To examine if *Sl*SCPx protein can influence the cholesterol uptake, cholesterol levels in the transfected cells were measured and compared with that in the control cells. The intact *Sl*SCPx coding region and *Sl*SCPx-2 domain region alone were cloned, respectively, into the pEGFP-1 vector replacing the GFP and under the control of the insect baculovirus IE1 promoter. The recombinant plasmids were used to transfect Spli-221 cells to express these recombinant proteins. After overnight culture in sterol-free medium, the medium was replaced with fresh medium containing 0.2 mg cholesterol per ml of medium. The cells were collected after overnight culture in this sterol medium. Protein expression and cholesterol levels in the cell extracts were analyzed. The anti-*Sl*SCPx antibody detected 58-kDa and 16-kDa bands in the protein extracts from the *Sl*SCPx- and *Sl*SCPx-2-transfected cells, respectively (Fig. 7A), indicating that the recombinant proteins (58 kDa for *Sl*SCPx and 16 kDa for *Sl*SCPx-2) were expressed in the transfected cells. An additional protein band of approximately 50-kDa immunologically cross-reacted with the antibody in all of the transfected cells (including the pEGFP-1 control) and needs to be identified. HPLC analysis of cholesterol levels in the transfected cells revealed that the *Sl*SCPx and *Sl*SCPx-2 transfected cells contained higher levels of cholesterol than the control cells transfected with the pEGFP-1 vector (Fig. 7B). The effect of *Sl*SCPx-2 appeared to be more significant (p < 0.01) than *Sl*SCPx (p < 0.05) on the increase of the cholesterol level as compared to the control. This resulted suggests that expression of *Sl*SCPx or *Sl*SCPx-2 increased cholesterol uptake into the Spli-221 cells from the sterol-containing medium.

### Effects of dsRNA interference of *Sl*SCPx transcripts on cholesterol uptake in larvae

**Figure 8:**
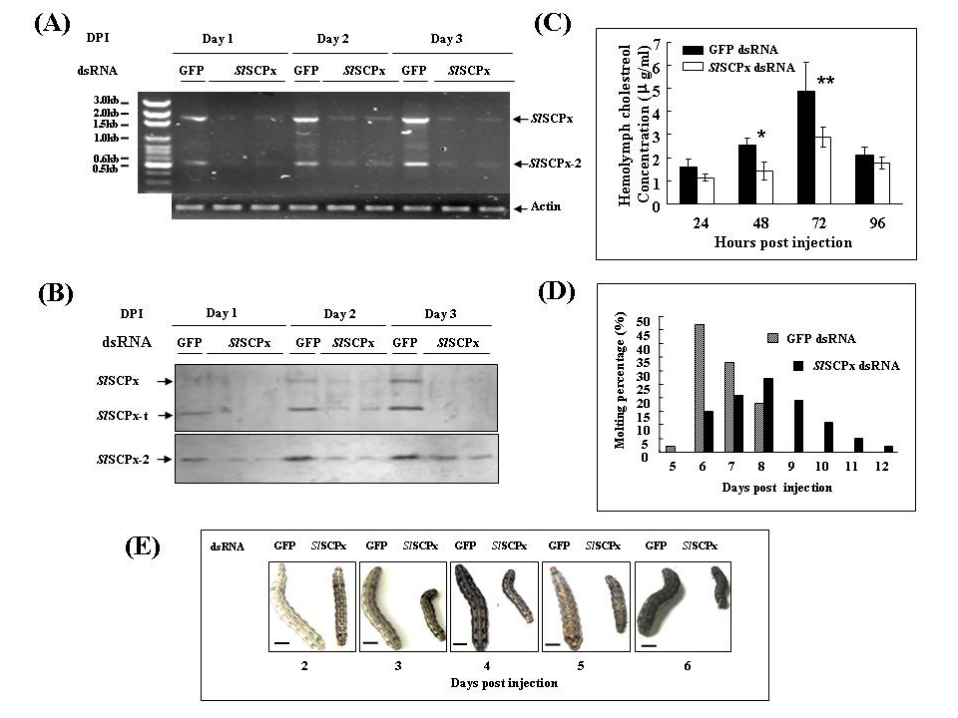
**Effects of *Sl*SCPx dsRNA interference on the levels of the transcripts and proteins of the *Sl*SCPx gene, cholesterol level in the hemolymph and molting and growth of the 6^th ^instar larvae**. (A) A total of 4 μg of dsRNA per larva was injected into the larvae after ecdysis into the 6^th ^instar stage. Total RNA and proteins were extracted from the midgut of the 30 treated larvae for transcript analysis by RT-PCR and protein analysis by western blotting analysis, respectively. In the RT-PCR analysis, α-actin amplification from the same RNA samples as the target gene was used as an internal control. DPI: days post injection. (B) In the western blotting analysis 30 μg of proteins was loaded per lane and were probed with anti-*Sl*SCPx antibody (upper panel of B) and anti-*Sl*SCPx-2 antibody (lower panel of B), respectively. For the *Sl*SCPx treatments, expression of RNA (A) and protein (B) in two independent individual samples are shown. The control was the samples injected with GFP dsRNA (A and B). (C) The hemolymph cholesterol levels in the *Sl*SCPx dsRNAi treated insects and the control. Three independent replicates were performed for each data point. The concentration of cholesterol in the cells or hemolymph was calculated as μg/ml of medium or hemolymph. (D) Pupation incidences in dsRNA-treated larvae. The scale bars represent 8 mm.

To examine the function of *Sl*SCPx, dsRNA of *Sl*SCPx was synthesized *in vitro *and injected into the hemolymph of larvae molting from 5^th ^to 6^th ^instar stage. RT-PCR and western blotting analyses indicated that the *Sl*SCPx dsRNA efficiently reduced the levels of both the *Sl*SCPx and *Sl*SCPx-2 transcripts (Fig. 8A) and the three proteins (Fig. 8B) of the target *Sl*SCPx gene between day 1 and 3 post injection. The hemolymph was collected after 24, 48, 72 and 96 h post injection and cholesterol levels in the hemolymph were examined by using HPLC. The results indicated that *Sl*SCPx-dsRNA-treated larvae had a significant decrease in the hemolymph cholesterol level at day 2 (p < 0.05) and day 3 (p < 0.01) post injection, as compared to the GFP-dsRNA-treated control larvae (Fig. 8C). Furthermore *Sl*SCPx dsRNA interference resulted in a delay in the larval development, for example, from the larval to the pupal transformation by an extension of 2-3 days as compared to the controls (Fig. 8D). All of the control larvae completed the molting process before day 9 post injection, whereas more than 40% of the *Sl*SCPx-dsRNA-treated larvae were still at the last larval stage until day 12 post injection. The *Sl*SCPx-dsRNA-treated larvae were smaller in body size than the control animals (Fig. 8E). The results from the RNAi-treatment suggest that knocking down the transcripts of *Sl*SCPx reduced the level of cholesterol in the hemolymph and retarded the growth and development of the larvae, probably by affecting the synthesis of ecdysteroid and cell membrane formation among other things.

## Discussion

The SCP-2 gene family has been implicated in the absorption and transport of lipids, fatty acids and cholesterols [1]. Studies have shown that SCP-2 proteins have multiple functions because of their capability to bind with cholesterol, fatty acids and fatty acyl CoA and regulating lipid rafts and signalling processes [28-32]. The human SCPx gene, which is a member of the SCP-2 family, encodes a 58-kDa fusion protein that is post-translationally processed into a 46-kDa thiolase and a 13-kDa SCP-2 protein. Only one single copy of the SCPx/SCP-2 gene is found in the genomes of humans, mice and rats [1]. In *C. elegans *the homologous gene encodes a P44 protein, which lacks the SCP-2 domain at the 3'-terminal region of the protein [18]. In Diptera, such as *D. melanogaster *and *A. aegypti*, SCPx gene produces a single transcript encoding a full-length SCPx, but does not encode SCP-2 mRNA [19,20]. Instead, separate genes in the *A. aegypti *genome encode small SCP-2 and SCP-2-like proteins, which do not contain the thiolase domain [33,34]. In lepidopteran insects, such as *S. littoralis *[21] and *B. mori *[22], which is a very close allopatric species of *S. litura*, a single SCPx/SCP-2 gene encodes both SCPx and SCP-2 mRNAs, which suggests that it is generated by alternative splicing into two mRNA isoforms after transcription and no other separate SCP-2 genes for SCP-2 proteins are present in these lepidopteran genomes. In this study, northern blotting analysis detected three transcripts of 2.8 kb (putative pro-*Sl*SCPx), 2.0 kb (*Sl*SCPx) and 0.9 kb (*Sl*SCPx-2) in the midgut of *S. litura *when a DNA fragment of the SCP-2 coding region (*Sl*SCPx-2) of *Sl*SCPx was used as a probe (Fig. 5A). However, when the *Sl*SCPx-t region of the *Sl*SCPx gene was used as a probe, the 0.9 kb band was not detected (Fig. 5C). RT-PCR using a pair of primers specific to the 5'-terminus and 3'-teminus of the full-length SCPx ORF and total RNA isolated from the larval tissues amplified two cDNAs that encoded for the full-length *Sl*SCPx mRNA and the *Sl*SCPx-2 domain mRNA, respectively (Fig. 2). There are at least three possible explanations for the multiple transcripts of *Sl*SCPx gene: One is that the two amplified mRNA transcripts are the products of the same *Sl*SCPx gene, but result from different transcription initiation sites, as is the case in human SCPx/SCP2 gene [9]. In humans, the SCPx gene generates a 2.8-kb mRNA encoding a fused 58-kDa protein (3-ketoacyl-CoA thiolase plus SCP-2) and a 1.5-kb mRNA encoding only a 15.3-kDa SCP-2 protein [9]. The 58-kDa protein was post-translationally cleaved into 46-kDa thiolase and 15-kDa SCP-2 proteins [1]. The 15-kDa pro-SCP-2 protein is the precursor of the mature 13-kDa SCP-2 and the 20-amino acid peptide at the 5'-terminal end may alter SCP-2 structure to facilitate peroxisomal targeting [28,29]. However, in the present study the promoter sequence of the gene was not determined and the genomic organization of the gene has not been elucidated, therefore we do not know if there was any promoter regulation of the gene to provide two transcripts. A second possibility is that there are other separate genes that encode SCP-2 mRNA transcripts in the midgut. However, Southern blotting analysis of genomic DNA indicated that only a single band was detected (data not shown), suggesting that only one gene of *Sl*SCPx is present in the genome. Thirdly, perhaps two transcripts were derived from alternative splicing of a full-length transcript (*Sl*SCPx) into the two transcripts post transcriptionally, encoding the full-length *Sl*SCPx and *Sl*SCPx-2, respectively, as suggested by the two full-length *Sl*SCPx mRNA and *Sl*SCPx-2 mRNA amplified PCR products detected (Fig. 2A). Because the primers used would be unable to detect a transcript for the *Sl*SCPx-t region only we cannot rule out such a transcript. Nevertheless, northern blotting analyses revealed that no mRNA transcript for *Sl*SCPx-t only, which was expected to be 1.23 kb, was detected when either *Sl*SCPx or *Sl*SCPx-t DNA fragment was used as a probe. Therefore, the most likely explanation is that the full-length transcript (*Sl*SCPx) was alternatively spliced into the two transcripts post transcriptionally. Our hypothesis concurs with the demonstration in *B. mori*, where the BmSCPx/SCP-2 gene also contains thiolase and SCP-2 domains and they are encoded by two separate exons, respectively [22], generating two isoforms of SCPx and SCP-2 mRNA. This is also similar to the cases in mammals [1] but differs from the Diptera, such as *D. melanogaster *and *A. aegypti*, in which the SCPx gene generates only SCPx mRNA but not SCP-2 mRNA [19,20]. These results and analyses suggest a hypothesis that the *S. litura Sl*SCPx gene produces an mRNA transcript that contains both a *Sl*SCPx-t domain for a 3-oxoacyl-CoA thiolase and a *Sl*SCPx-2 domain for a SCP-2 protein, and a *Sl*SCPx-2 mRNA transcript for *Sl*SCP-2 protein by splicing (Fig. 2). It is interesting that in the feeding larvae the *Sl*SCP-2 mRNA was found not only in the midgut but also in the epidermis and fat body, where no *Sl*SCPx mRNA was detected. This suggests that this alternative transcription of *Sl*SCx-2 can occur in different tissues other than the midgut. However, although there was *Sl*SCPx-2 transcripts detected in the epidermis of feeding larvae, for example at day 3 of 6^th ^instar stage (Fig. 5A), but no immunoreactive protein of *Sl*SCPx-2 was detected by western blotting analysis. This probably indicates that only a low level of *Sl*SCPx-2 protein translation occurred in the epidermis at this stage, although the *Sl*SCPx-2 transcripts were present. The regulatory mechanism for transcription and translation of the *Sl*SCPx-2 gene is worth to be further examined.

In addition to the regulation of gene expression at the transcription level, the regulation of the protein production can take place at the post-translational level. The anti-*Sl*SCPx-t antibody detected two protein products of 58 kDa (for full-length *Sl*SCPx) and 44 kDa (for *Sl*SCPx-t) in the midgut (Fig. 5E and 5F), while the anti-*Sl*SCPx-2 antibody detected a product of 16 kDa (for *Sl*SCPx-2) in the midgut and fat body (Fig. 5D and 5F). These data suggest that the *Sl*SCPx gene is transcribed into a full-length mRNA that is translated into a polypeptide, which is proteolytically cleaved into two peptides: 3-oxoacyl-CoA thiolase (*Sl*SCPx-t) and *Sl*SCPx-2, through a post translational activity. It was also observed that the *Sl*SCPx protein was cleaved into thiolase and SCP-2 at a much higher level (Fig. 5F) than the vertebrate SCPx which is processed into the thiolase domain and SCP-2 domain proteins post-translationally at about 50% level [1].

In mouse, knocking out the SCPx/SCP-2 gene, which affect both SCPx and SCP-2 expression, showed that this gene is required for peroxisomal β-oxidation of methyl-branched fatty acyl-CoAs and bile acid precursors [13]. In human, SCPx mutation, which only affected SCPx but not SCP-2 expression, resulted in an accumulation of the branched-chain fatty acid, pristanic acid, and excretion of abnormal bile alcohol glucuronides in urine [32]. These results indicate that the SCPx gene plays critical roles in the breakdown of branched-chain fatty acids and bile acid metabolism. Some other studies have revealed that SCPx/SCP-2 and SCP-2 proteins are involved in cholesterol binding and transport [14,34-36]. Three lines of evidence show that both of the *Sl*SCPx and *Sl*SCPx-2 proteins play critical roles in cholesterol absorption in *S. litura*. Firstly, northern and western blotting analyses showed that the *Sl*SCPx mRNA was specifically present at higher levels in the midgut of the feeding 6^th ^larvae (Fig. 5A and 5C), while the *Sl*SCPx-2 mRNA was predominately present in the midgut, but also in both the fat body and epidermis (Fig. 5B). The expression of *Sl*SCPx mRNA was much higher during the feeding stages than the non-feeding stages, indicating that these proteins may be necessary for absorption and transport of nutrients such as cholesterol, during larval feeding stages. Secondly, the over-expression of *Sl*SCPx and *Sl*SCPx-2 proteins increased cholesterol uptake into the *in vitro *cultured *S. litura *Spli-221 cells from the culture medium (Fig. 7). Thirdly, when the *Sl*SCPx mRNA was suppressed by *Sl*SCPx dsRNA interference the hemolymph cholesterol level in the treated larvae decreased and the development of the insect was delayed (Fig. 8). Collectively these results suggest that *Sl*SCPx and *Sl*SCPx-2 may play critical roles in cholesterol absorption in *S. litura*.

This study provides evidence for the association or links among SCPx gene, cholesterol accumulation and development in lepidopteran insects. Insects must take up cholesterol from their diet and accumulate the cholesterol in their bodies during the feeding stages for their growth and development because they lack key cholesterol biosynthesis enzymes, such as squalene monooxygenase, and lanosterol synthase [3,37]. The midgut and possibly the foregut are the sites of cholesterol absorption [38-40] and the midgut is a major site for cholesterol absorption into the hemolymph [39]. It has also been demonstrated that SCP-2 protein is necessary for cholesterol uptake in *A. aegypti *[24]. Knocking down AeSCP2 transcript expression in *A. aegypti *resulted in a reduction of cholesterol accumulation in pupae and a high mortality in developing adults and reduced egg viability [24]. It is well known that cholesterol is a precursor of 20-hydroxyecdysone (20E) and is a structural component of cell membranes [3]. When cholesterol is absent from the diet, the *Heliothis zea *neonate larvae fail to molt into 2^nd ^instar stage or suffer a delay in larval growth and pupation [41,42]. The present study demonstrates an association or link between SCPx gene, cholesterol accumulation and larval development. Like AeSCP2 in *A. aegypti *[24], interference in expression of the *Sl*SCPx transcript in the larvae that just molted into 6^th ^instar stage resulted in a decrease in the cholesterol accumulation in the hemolymph and a delay in the larval development and pupation (Fig. 8C-E). Since cholesterol is a precursor of 20E and is a structural component of cell membranes [3], inhibition of uptake and transport of cholesterol by *Sl*SCPx RNAi would impacts adversely on the biosynthesis of 20E that initiates and regulates molting and metamorphosis, resulting in a delay in larval development and pupation. However, in this study only hemoplymph cholesterol levels were examined and the cholesterol levels in the fat body and midgut as well as other tissues were not determined. Whether or not the changes in the hemoplymph cholesterol levels are due to the release of cholesterol from other tissues such as the fat body need to be examined. Direct evidence for the 20E level alteration by the *Sl*SCPx suppression and cholesterol accumulation will help define the relationships among these three processes. Another possible mechanism for the inhibition of larval growth and metamorphosis by *Sl*SCPx suppression is that these proteins may be involved in fatty acid metabolism [28,29] and signal transduction [31] and may have to be examined. Since *Sl*SCPx dsRNA covering the *Sl*SCPx-t and *Sl*SCPx-2 domains (Fig. 2) were used to inhibit the *Sl*SCPx transcription and it appeared that both of *Sl*SCPx and *Sl*SCPx-2 transcripts were efficiently knocked down (Fig. 8A and 8B), and it is not clear which protein, if either plays a more critical role in cholesterol accumulation, ecdysteroid synthesis and larval-pupal transformation. We have laid the groundwork for further studies to critically examine the different possibilities.

In vertebrates, although amino acid sequences of the *C*-terminal region (SCP-2 domain) of SCPx and other SCP-2 proteins are identical and have a peroxisomal localization signal sequence (AKL or SKL), SCPx locates exclusively in peroxisomes, while SCP-2 is found in both peroxisomes and cytosol [1]. In *A. aegypti*, *Ae*SCPx is mostly restricted in the peroxisomes, whereas *Ae*SCP2 encoded by another independent *Ae*SCP2 gene was detected mostly in the cytosol [25]. A recent study indicates that the human SCPx protein undergoes post-translational processing on the peroxisomal surface, releasing a short *C*-terminal product (SCP-2) that acts as a transcription factor [14]. It has been reported that SCPx/SCP-2 is regulated through the Forkhead transcription factor FOXO3a and SCP-2 is speculated to protect fatty acids from peroxidation [43]. The preliminary results of the present study indicate that *Sl*SCPx, *Sl*SCPx-t and *Sl*SCPx-2 proteins were distinct in their subcellular distribution. In the transfected Spli-221 cells, *Sl*SCPx and *Sl*SCPx-t proteins were present mostly in the cytoplasm (Fig. 6B, Dc and 6De), while *Sl*SCPx-2 was localized in both the nuclei and cytoplasm, with a slightly higher level in the nuclei than in the cytoplasm (Fig. 6C and 6Dg). But it remains unclear whether or not *Sl*SCPx-2 in nuclei acts as a transcription factor. Interestingly, it was found that the *Sl*SCPx, *Sl*SCPx-t and *Sl*SCPx-2 proteins had different distributions in the midgut epithelium of the feeding larvae (Fig. 6Ab-d). *Sl*SCPx protein (or *Sl*SCPx-t plus *Sl*SCPx-2 proteins) was detected by the anti-*Sl*SCPx antibody, which is expected to immunologically react with both of *Sl*SCPx-t and *Sl*SCPx-2 proteins, throughout the epithelial cells (Fig. 6Ac), while the *Sl*SCPx-t protein detected by the specific anti-*Sl*SCPx-t antibody was distributed predominately in the epithelial cells that were close to the lumen side (Fig. 6Ad) and the *Sl*SCPx-2 protein detected by the specific anti-*Sl*SCPx-2 antibody was localized in the cells that were close to the basal membrane side of the midgut epithelium (Fig. 6Ae). These observations may indicate that *Sl*SCPx-t and *Sl*SCPx-2 proteins play different roles in cholesterol uptake and transport in the midgut of *S. litura*. In the *in vitro *transfection experiments using Spli-221 cells, the *Sl*SCPx/GFP fusion proteins contained a GFP peptide. It is not clear if this extra peptide can interfere with the accurate localization, although many reports in the literature indicate that this technique has been used to determine the localization of target genes. Higher resolution is critical to unambiguously establish the sub-cellular localization which should be done in subsequent study. Having established the characterization of the gene and the broad functional analyses we are now in a position to unravel the mechanism behind our observation.

## Conclusion

Cholesterol is required for cell membranes and ecdysteroid biosynthesis in insects. Ecdysteroids regulate growth, development and reproduction in insects. However, insects cannot synthesize cholesterol through *de novo *biosynthesis and therefore insects must obtain cholesterol or sterols from their diet to fulfill the requirements for their normal growth, development and reproduction. In the present study, by analyzing the gene expression patterns, protein localization, protein over-expression and transcript interference we demonstrated that the *Sl*SCPx gene was expressed predominately in the midgut of larvae at the feeding stages and was involved in cholesterol uptake and normal development in *S. litura*. Interference of this gene resulted in a decrease in cholesterol uptake, which may result in inhibition of ecdysteroid biosynthesis and consequently a delay in the larval-pupal metamorphosis. Besides understanding the mechanism of cholesterol uptake this study might provide a potential molecular target for insect pest management.

## Methods

### Experimental insects and cells

*Spodoptera litura *Fabricius (Lepidoptera: Noctuidae) and Spli-221 cell line were from The Entomology Institute of SUN YAT-SEN University, Guangzhou, China. Larvae were reared an artificial diet at 26°C in 70-80% humidity and a photoperiod of 12 h light and 12 h dark until they reached the pupal stage or became adult moths. The artificial diet used for rearing the insect contained 100 g soybean powder, 80 g wheat bran, 26 g yeast, 8 g casein, 8 g Vitamin C, 1 g choline chloride, 2 g sorbate, 0.2 g cholesterol, 0.2 g inositol, 26 g agar and 2 ml formaldehyde in one liter. The Spli-221 cell line was cultured at 28°C in Grace's insect medium (Invirogen Co., Guagnzhou, Guangdong, China) containing 10% fetal bovine serum. Cells were passaged every 4 days using a 1:4 dilution of cells.

### Chemicals and materials

DNA restriction enzymes, T4 ligase, Taq RNA polymerase and Random Primer Labelling Kit were purchased from TAKALA BIO Inc (Otsu, Shiga, Japan). Sheep anti-rabbit IgG-SABC-FITC (Strept Avidin-Biotin Complex-fluorescein isothiocyanate) and cholesterol (chromatographic grade) were purchased from Sigma-Aldrich Co. (Guangzhou, Guangdong, China). Trizol-reagent for RNA extraction, lipofectin and Grace's insect medium were purchased from Invitrogen Co. (Guangzhou, Guangdong, China). The steroid-free medium was made according to the description by Lan et al. [44]. MEGAscrip RNAi Kit (Silencer™) for double strands RNA synthesis was purchased from Ambion (Austin, USA). Gel DNA Extraction Kit and Plasmid DNA Preparation Kit were purchased from Qiagen (Pudong, Shanghai, China). Acetonitrile, isopropanol and methanol were chromatographic grade and purchased from Shanghai Biotechnology Inc. of China. Northern blotting membranes (Hybond-XL) and western blotting membranes (Hybond-C) were purchased from Amersham Bioscience Inc. (Piscataway, NJ, USA). His Bind Columns for His-tag protein purification was purchased from EMD Chemicals Inc. (Darmstadt, Germany). pEGFP-1 plasmid DNA was from Clontech Laboratories Inc. (Mountain View, CA, USA). The pPROEXTM HTa expression vector was from Life Technologies Inc. (Gaithersburg, MD, USA). Bradford Protein Assay Kit was purchased from Bio-Rad Laboratories (Hercules, USA).

### Sequence analysis

Annotation was performed using the Alignment Search tools in the National Center for Biotechnology Information BLAST search services [45]. Comparison, alignment and phyologenetic tree analysis of multiple sequences were conducted using CLUSTAL W in MegAlign 5.01 [46] of DNASTAR (DNASTAR, Inc., Madison, WI) at a Gap Penalty of 10 and a Gap Length Penalty of 0.2.

### RNA isolation and northern blot analysis

Total RNA was isolated from larval tissues using Trizol-reagent (Invitrogen, Guangzhou, Guangdong, China). The fat body, midgut and epidermis were dissected from the larvae of 4^th ^to 6^th ^instar stages and pupae and immediately frozen in liquid nitrogen, and then stored at -80°C until RNA isolation. Total RNA (10 μg) per sample was separated on 1.0% formaldehyde agarose gels and transferred onto nylon membranes. *Sl*SCPx cDNA was labelled with ^32^P-dCTP using the Random Primer DNA Labelling Kit (TAKALA BIO. Inc., Otsu, Shiga, Japan) and used as probes for northern blot analysis. Pre-hybridization, hybridization and post-hybridization washes were carried out according to Beliveau *et al. *[47]. Membranes were scanned and photographs were taken in Typhoon TRIO Variable Mode Imager (Typhoon 9400, GE Healthcare Life Sciences, USA).

### *In vitro *expression and purification of *Sl*SCPx, *Sl*SCPx-t and *Sl*SCPx-2 proteins

For recombinant protein expression in bacterial, the open reading frames (ORFs) of the *Sl*SCPx, *Sl*SCPx-t and *Sl*SCPx-2 cDNAs were amplified by PCR and inserted into the pPROEXTM HTa expression vector (Life Technologies, Burlington, Canada) with 6× His tag on the C-terminal ends of the target sequences. *E. coli *cells (DH-5α) were transformed with the recombinant plasmid DNAs (pPROEXTM HTa-*Sl*SCPx, pPROEXTM HTa-*Sl*SCPx-t and pPROEXTM HTa-*Sl*SCPx-2). Expression of the His-tagged *Sl*SCPx, *Sl*SCPx-t and *Sl*SCPx-2 fusion proteins was induced by adding IPTG (isopropyl-β-D-Thiogalac-topyranoside) at a final concentration of 1 mM. The recombinant proteins were purified using His-tag affinity columns (EMD Biosciences, Darmstadt, Germany). Protein concentrations were determined using Bradford Protein Assay Kit (Bio-Rad, Hercules, USA).

### Production of *Sl*SCP-x, *Sl*SCPx-t and *Sl*SCP x-2 antibodies

The purified recombinant *Sl*SCPx, *Sl*SCPx-t and *Sl*SCPx-2 proteins were separated on SDS-PAGE gels and stained with Coomassie Blue R-250 and the target bands were excised. The proteins were mixed with Freund's adjuvant and injected into New Zealand White rabbits. Antisera were collected after three booster injections, each with 500 μg of the recombinant proteins. Pre-immune serum collected from the same rabbit prior to immunization was used as a control.

### SDS-PAGE and western blotting analysis

Insect tissues were homogenized in lysis buffer (0.25 M Tris-HCl, pH 8.0, 0.2% Triton X-100, 1 mM dithioerythritol, 5 mM EDTA, 10 mM β-mercaptoethanol, 1 mM phenylmethylsulphonyl fluoride, protease inhibitor cocktail), and centrifuged at 12,000 × g at 4°C for 15 min. Supernatants containing soluble proteins were stored at -80°C. Protein samples were denatured at 100°C for 5 min in an equal volume of 2× protein loading buffer (0.1 M Tris buffer, pH 6.8, 4% SDS, 0.2% β-mercaptoethanol, 40% glycerol, and 0.002% bromophenol blue). Protein concentrations were determined using Bradford Protein Assay Kit (Bio-Rad, Hercules, USA). SDS-PAGE was performed in 12%-15% acrylamide gels in tris-glycine-SDS buffer (10 mM Tris, 50 mM glycine, 0.1% SDS, pH 8.0). The gel was stained with Coomassie Blue R-250. The molecular mass of the proteins was calculated using the GeneTools program Tiangen (Tiangen Biotech, Beijing, China). For western blotting analysis, proteins were transferred from the acrylamide gels to nitrocellulose membranes. The membranes were blocked with 3% BSA in 1× PBS buffer for 2 h at room temperature, and then incubated with the *Sl*SCPx, *Sl*SCPx-t and *Sl*SCPx-2 antibodies (1:1,000) at room temperature for 1 h. Goat anti-rabbit IgG (Dingguo Biotechnology, Beijing, China) conjugated with alkaline phosphatase was used as the secondary antibody at a dilution of 1:2,000. Nitroblue tetrazolium and 5-bromo-4-chloro-3-indolyl phosphate was used as substrates for color development.

### Immunohistochemistry

Immunohistochemistry localization of *Sl*SCPx, *Sl*SCPx-t and *Sl*SCPx-2 were performed as described by Feng *et al*. [48]. Whole larvae at the selected stages were fixed with 4% formaldehyde in 1× PBS buffer for 24 h at 4°C and embedded in paraffin. Eight-micrometer thick sections were made for immunostaining. The sections were stained first with the primary antibody at a dilution of 1:200 for 1 h. The secondary antibodies, sheep anti-rabbit IgG conjugated with SABC-FITC (Strept Avidin-Biotin Complex-fluorescein isothiocyanate) (Sigma, Guangzhou, China), were used according to the manufacturer's protocol at a dilution of 1:100. The sections were counter-stained with 4', 6-diamidine-2'-phenylindole dihydrochloride (DAPI) for 30 min and examined under a fluorescence microscope (DMI4000B). The photographs were taken as double exposures using fluorescein and DAPI filters.

### Preparation of cytoplasmic and nuclear proteins

Cytoplasmic extracts were prepared as described by Ko and Puglielli [14] with some modifications. Midgut tissues were homogenized in homogenization buffer containing 25 mM Tris-HCl, pH 7.4, 0.5 mM EDTA, 0.5 mM EGTA and a protease inhibitor mixture. The homogenates were centrifuged first at 500 × g for 5 min. The supernatant were collected and centrifuged at 14,000 × g for 15 min and the supernatants containing cytoplasmic proteins were collected.

For nuclear extraction, midgut tissues were suspended in 3 volumes of lysis buffer (20 mM Hepes, pH 7.9, 10 mM KCl, 1 mM EDTA, pH 8.0, 10% glycerol and 10% protease inhibitor mixture), followed by incubation on ice for 30 min. The resultant supernatants were gently agitated by gently pipetting in and out; the lysates were then centrifuged at 14,000 g for 30 min at 4°C to obtain nuclear pellets. Nuclear pellets were washed twice with cell lysis buffer (lacking protease inhibitor mixture) and then re-suspended in 2 volumes of nuclear extract buffer (20 mM HEPES, pH 7.9, 10 mM KCl, 1 mM EDTA, pH 8.0, 420 mM NaCl, 20% glycerol, and 10% protease inhibitor mixture). The nuclear proteins were extracted by incubation at 4°C for 30 min with gentle agitation, followed by centrifugation at 14,000 g at 4°C for 30 min. The resultant supernatant fraction was used as a nuclear extract.

### Expression of *Sl*SCPx/GFP, *Sl*SCPx-t/GFP and *Sl*SCPx-2/GFP fusion proteins in Spli-221 cell line

To examine the intracellular location of the *Sl*SCPx, *Sl*SCPx-t and *Sl*SCPx-2 proteins in Spli-221 cells, SCP/GFP fusion constructs (*Sl*SCPx/GFP, *Sl*SCPx-t/GFP and *Sl*SCPx-2/GFP) were made with the transfection vector pEGFP-1 (Clontech Laboratories Inc., Mountain View, CA, USA) with the GFP fragment at the *C*-terminal end of the fusion proteins. Spli-221 cells were seeded at 2 × 10^5 ^cells/ml in 2 ml of Grace's medium in 6-well plates. After overnight incubation, the medium was removed and the cells were washed once with 2 ml of the transfection medium without serum and antibiotics. One milliliter of the transfection medium containing 4 μg/ml transfection vectors and 20 μg/ml lipofectin (Invitrogen Co., Guangzhou, Guangdong, China) was added to the cell cultures. The cells were incubated in the transfection medium for 8 h and then the transfection medium was replaced with 2 ml of fresh Grace's insect medium containing 10% fetal bovine serum (Invitrogen Co., Guangzhou, Guangdong, China) and incubated at 28°C. After 24 h culture, the transfected cells were observed for green fluorescence signals and photographed by laser confocal microscope.

### Expression of *Sl*SCPx, and *Sl*SCPx-2 in Spli-221 cell line

To examine if *Sl*SCPx and *Sl*SCPx-2 proteins can influence the cholesterol uptake, the intact *Sl*SCPx coding region and *Sl*SCPx-2 domain region alone were cloned, respectively, into the pEGFP-1 vector replacing the GFP and under the control of the IE1 promoter. The cholesterol level in the transfected cells was measured and compared with the level in the control cells. Spli-221 cells were seeded at 2 × 10^5^cells/ml in 2 ml of Grace's medium in 35 × 10 mm culture dishes. After overnight incubation, the medium was removed and the cells were washed once with 2 ml of the transfection medium without serum and antibiotics. One milliliter of the transfection medium containing 4 μg/ml *Sl*SCPx and *Sl*SCPx-2 expression vectors and 20 μg/ml lipofectin was added into the cell cultures. The cells were incubated in the transfection medium for 8 h and then the transfection medium was replaced with 2 ml of fresh steroid-free medium containing 10% fetal bovine serum and incubated at 28°C. After overnight culture in the steroid-free medium, the medium was replaced with the same medium but containing 0.2 mg cholesterol/ml and the cells were cultured for an additional 12 h (25, 44). The cells were washed twice with 2 ml cold 1× PBS and the total cellular lipids were extracted as described below according to Moncecchi et al.[36] for protein and cholesterol analysis.

### Cholesterol analysis by high performance liquid chromatography (HPLC)

Extraction of lipids and sterols from the cell line were conducted as described below. At the indicated time intervals, medium was removed quickly, the cells were washed twice with 1× PBS, and frozen in liquid N_2_. The lipids were extracted by adding a mixture of chloroform: methanol 2:1 (v/v) to the collected cells. The samples were mixed well by inverting the tube several times, and then centrifuged at 12,000 *g *for 20 min at 4°C. The supernatant was collected and a mixture of chloroform:methanol 2:1 (v/v) was added to the pellet to extract the lipids. This step was repeated three times. The supernatants were collected, pooled and evaporated to dryness under a stream of nitrogen. The pellet was dissolved in 200 μl methanol and the proteins were filtered through a filter membrane (0.45 μm) and then stored at -70°C until use. Cholesterol concentrations were determined by HPLC using a ZORBAX XDB C-18 column, 4.65 μm × 250 mm. under the following conditions: mobile phase:isopropyl alcohol:acetonitrile 4:1 (v/v); flow rate:1.2 ml/min; wavelength: 208 nm; injection volume: 20 μl; column temperature: 22°C.

For hemolymph cholesterol extraction and determination, the process was the same as for the cells, except that the washing step using PBS was skipped. The hemolymph was collected from three larvae for each time point of the treatments and control. Three independent replicates were utilized for each data point. The significance analysis of the differences between the treatments and the control were performed using ANOVA followed by Duncan's Multiple Comparison Test. The data represent mean ± SD, n = 3. Asterisks "**" and "*" indicate significance at p < 0.01 and p < 0.05 levels, respectively. The concentration of cholesterol in the cells or hemolymph was calculated as μg/ml of medium or hemolymph.

### Double stranded RNA synthesis and RNA interference

High quality dsRNA was generated using a MEGAscript RNAi Kit (Silencer™, Ambion, Austin, USA) according the manufacturer's instructions. A twenty base T7 promoter sequence was added to the sense and antisense target sequences: the sense 5'-**TAATACGACTCACTATAGGG**CTGCTGTGTTTGAGGAGAA-3', the antisense 5'-**TAATACGACTCACTATAGGG**ACAAACTAGTACCAGTTTACAA-3' (The underlined bases represent T7 promoter sequence). The quantity of *Sl*SCPx dsRNA was determined by NanoDrop Spectrophotometer measurement at 260 nm and by agarose gel analysis. The dsRNA was dissolved in elution buffer (10 mM Tris-HCl, pH 7, 10 mM EDTA) and used for injection. Double stranded RNA of green fluorescence protein (GFP) was made using the same method and used as negative control.

Larvae that had just molted into the 6^th ^instar stage displaying a white head capsule were used for dsRNA injection. The larvae were anesthetized on ice for 5 min prior to microinjection. Four micrograms per larva of dsRNA were injected into the larvae at the intersegment behind the second abdominal segment using a microinjector. Backflow of the body fluid was avoided by sealing with Vaseline and the larvae that lost too much body fluid were discarded. Sixty larvae in each of the three replicates were injected for each of the treatment groups. The larvae were then returned to the artificial diet and reared at 26°C until they became adults or were sacrificed for sample collection for RNA, protein and cholesterol analysis. The midgut and hemolymph were collected from 30 individual larvae for RNA and protein analysis and for cholesterol analysis, respectively. Each of the midgut was cut into two equal portions, one for RNA extraction and the other for protein extraction. The remaining 30 animals were then reared for development observation. For cholesterol analysis, the data were represented as mean ± standard deviation and each data point was generated from the hemolymph from three larvae in each of the three replicates.

## Authors' contributions

XRG performed gene cloning and characterization, *in vitro *protein expression, northern and western blotting analyses, immuohistochemistry, cholesterol HPLC analysis and RNAi analysis. SCZ and LL coordinated the project and assisted in protein expression, immunohistochemistry and cell culture. QLF and XRG designed the experiments and wrote the paper. QLF provided financial support. All authors have read and approved the final manuscript.
